# 2137 Percentage of viable tumor Versus radiation treatment effect in surgical specimens is not associated with outcomes in recurrent glioblastoma

**DOI:** 10.1017/cts.2018.175

**Published:** 2018-11-21

**Authors:** Robert D. Schwab, Stephen Bagley, Zev Binder, Robert Lustig, Donald O’Rourke, Steven Brem, Arati S. Desai, MacLean Nasrallah

**Affiliations:** 1 University of Pennsylvania School of Medicine, Philadelphia, PA, USA; 2 Abramson Cancer Center, Perelman School of Medicine, University of Pennsylvania, Philadelphia, PA, USA; 3 Department of Neurosurgery, Perelman School of Medicine, University of Pennsylvania, Philadelphia, PA, USA; 4 Department of Radiation Oncology, Perelman School of Medicine, University of Pennsylvania, Philadelphia, PA, USA; 5 Department of Pathology and Laboratory Medicine, Perelman School of Medicine, University of Pennsylvania, Philadelphia, PA, USA

## Abstract

OBJECTIVES/SPECIFIC AIMS: In patients with recurrent glioblastoma (GBM) who undergo a second surgery following standard chemoradiotherapy, histopathologic examination of the resected tissue often reveals a combination of viable tumor and treatment-related inflammatory changes. However, it remains unclear whether the degree of viable tumor Versus “treatment effect” in these specimens impacts prognosis. We sought to determine whether the percentage of viable tumor Versus “treatment effect” in recurrent GBM surgical samples, as assessed by a trained neuropathologist and quantified on a continuous scale, is associated with overall survival. METHODS/STUDY POPULATION: We reviewed the records of 47 patients with histopathologically confirmed GBM who underwent surgical resection as the first therapeutic modality for suspected radiographic progression following standard radiation therapy and temozolomide. The percentage of viable tumor Versus “treatment effect” in each specimen was estimated by one neuropathologist who was blinded to patient outcomes. RESULTS/ANTICIPATED RESULTS: After adjusting for other known prognostic factors in a multivariate Cox proportional hazards model, there was no association between the degree of viable tumor and overall survival (HR 0.83; 95% CI, 0.20–3.4; *p*=0.20). DISCUSSION/SIGNIFICANCE OF IMPACT: These results suggest that, in patients who undergo resection for recurrent GBM following standard first-line chemoradiotherapy, histopathologic quantification of the degree of viable tumor Versus “treatment effect” present in the surgical specimen has limited prognostic influence and clinical utility.

